# Educational

**Published:** 1895-03

**Authors:** 


### EDUCATIONAL.

Harvard veterinary department has sixty-two students enrolled in her classes.

The return of Prof. Osler to McGill University, Montreal, Canada, means much to the veterinary department there. His interest in comparative medicine and the advancement of veterinary science will add an untold strength and be greatly appreciated by every true votary of the profession whose zealous eye watches every movement in educational circles.

We print in this number a truly suggestive and valuable letter from the pen of that venerable practitioner of veterinary science in Pennsylvania, Dr. Isaiah Michener. More than sixty years of active daily practice in the routine work of the profession have not lessened his ardor and interest in the true growth of the profession. Would that more of our younger generation in the calling had some of his zeal and enthusiasm in the work of upbuilding our profession.

Prof. R. S. Huidekoper is now engaged in delivering a special course of lectures embracing the following subjects: Physical Diagnosis, Examination of Special Parts, Emergency Treatment, Examination for Soundness, and Records and Reports, to veterinary students and practitioners in New York City. Such an opportunity is not often afforded the busy practitioner for brushing up on rusty points and keeping pace with the intellectual progress of the profession, and we feel sure it will be greatly appreciated by all those who may be so favored as to be able to attend.

The following changes have recently taken place in the teaching staff of the United States College of Veterinary Surgeons: Dr. J. H. Adamson, D.V.S., has been appointed to the Chair of Anatomy, and Prof. Charles M. Emmons, M.D., Ph.D., to the Chair of Physiology. The resignation of Prof. J. Ramsey Nevitt, M.D., was accepted, owing to the pressure of other work that forbids his giving the necessary time to instruction. Eleven students matriculated at the opening in October. The course is a graded one of three years, and all matriculants passed the examination as prescribed by the Association of Faculties of North America.

